# The role of tryptophan-AhR signaling in the pathogenesis of metabolic dysfunction-associated steatotic liver disease (MASLD): implications for therapeutic strategies

**DOI:** 10.3389/fimmu.2026.1806118

**Published:** 2026-03-18

**Authors:** Yinan Zhao, Shuhao Cheng, Jiu Liang, Guoying Yu

**Affiliations:** 1 Clinical Medical College of Qinghai University, Xining, China; 2 Department of Hepatology II, Fourth People’s Hospital of Qinghai Province, Xining, China

**Keywords:** AhR, immune modulation, inflammation, kynurenine, lipid metabolism, MASLD, therapeutic strategies, tryptophan metabolism

## Abstract

Metabolic dysfunction-associated steatotic liver disease (MASLD) has emerged as a significant global health concern, closely associated with metabolic syndrome and characterized by hepatic fat accumulation, inflammation, and fibrosis. While the pathogenesis of MASLD is multifactorial, recent research has highlighted the role of the tryptophan-aryl hydrocarbon receptor (AhR) signaling pathway in influencing both immune and metabolic functions in the liver. Tryptophan, an essential amino acid, is metabolized into various bioactive metabolites, such as kynurenine, that activate AhR. This activation modulates cellular processes including inflammation, oxidative stress, and lipid metabolism. Emerging evidence suggests that dysregulated tryptophan metabolism and AhR signaling contribute to the progression of MASLD, particularly through immune modulation and alterations in metabolic pathways. This perspective aims to provide an overview of the current understanding of tryptophan-AhR signaling in MASLD, discussing its potential as a therapeutic target and the challenges associated with targeting this pathway. Future research directions are proposed to explore how modulation of the tryptophan-AhR axis could offer novel therapeutic strategies for MASLD, providing new insights into its treatment and management.

## 1 Introduction

Metabolic dysfunction-associated steatotic liver disease (MASLD), previously referred to as non-alcoholic fatty liver disease (NAFLD), has rapidly emerged as a predominant cause of chronic liver disease on a global scale (1, 2). MASLD is intricately associated with metabolic syndrome, which includes conditions such as obesity, type 2 diabetes, dyslipidemia, and insulin resistance, all of which have witnessed a marked increase in global prevalence over recent decades (1–3). A recent meta-analysis has estimated that approximately 38% of the global population is affected by MASLD, representing a 50% increase over the past two decades and underscoring the escalating public health challenge posed by this disease (4). In addition to its high prevalence, MASLD has become a primary indication for liver transplantation in Western countries and significantly contributes to liver-related morbidity and mortality (5). Moreover, approximately 10–20% of individuals diagnosed with MASLD advance to metabolic dysfunction-associated steatohepatitis (MASH, previously known as non-alcoholic steatohepatitis or NASH), which is characterized by hepatocyte injury, inflammation, and an elevated risk of fibrosis, cirrhosis, and hepatocellular carcinoma (4, 6). The progression from simple fatty liver to more advanced stages underscores the need for early detection and effective therapeutic strategies to prevent disease advancement.

Despite its striking prevalence and clinical impact, the mechanisms underlying MASLD pathogenesis remain incompletely characterized (7). Among emerging pathways of interest is the tryptophan-aryl hydrocarbon receptor (AhR) signaling axis. Tryptophan, an essential amino acid, is metabolized by host and microbial enzymes into bioactive metabolites such as kynurenine, which can activate AhR, a ligand-activated transcription factor that regulates immune responses, oxidative stress, and lipid metabolism (8–10). Dysregulation of this pathway has been implicated in metabolic and inflammatory diseases, but the precise role of tryptophan-AhR signaling in MASLD pathophysiology requires further elucidation (11, 12).

This article will explore the following four critical questions:

1. How does AhR activation contribute to the inflammatory and metabolic changes observed in MASLD?
2. What role does AhR play in the crosstalk between liver immune cells and hepatic metabolism in MASLD?
3. How do tryptophan metabolites and AhR signaling influence lipid metabolism and fibrosis progression in MASLD?
4. What are the potential therapeutic strategies targeting the tryptophan-AhR signaling pathway for the treatment of MASLD?
5. What are the existing challenges and limitations in exploring the tryptophan AhR signaling pathway and its application in MASLD?

Exploring tryptophan-AhR signaling in MASLD offers promising insights into the disease’s pathogenesis and potential treatments. Despite progress, key questions about how tryptophan metabolites and AhR activation affect liver immune responses, lipid metabolism, and fibrosis in MASLD remain. Addressing these will enhance understanding and treatment options. This perspective reviews current knowledge on tryptophan-AhR signaling in MASLD, its therapeutic potential, and the need for further research to fully realize its clinical benefits, potentially leading to new treatments and better patient outcomes.

## 2 The role of tryptophan-AhR signaling and current research

**Figure 1:**
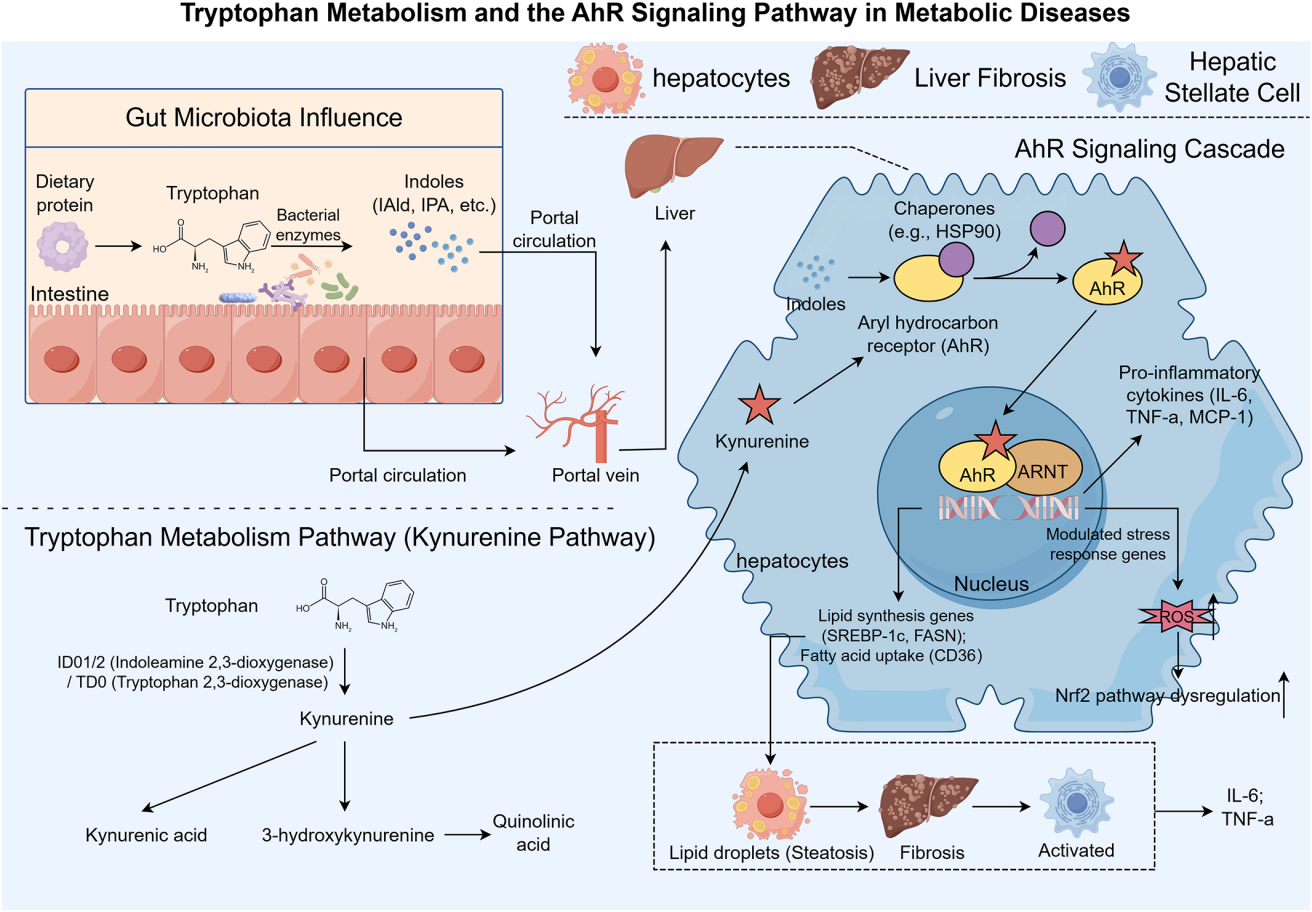
Schematic diagram of the tryptophan-kynurenine-AhR signaling axis in the pathogenesis of MASLD (this figure was created by Figdraw). Tryptophan, an essential amino acid derived from the diet, undergoes two major metabolic pathways to generate bioactive ligands for the aryl hydrocarbon receptor (AhR): (1) Gut microbiota-mediated metabolism produces indole derivatives (e.g., indole-3-acetaldehyde, indole-3-propionic acid), which enter the liver via the portal circulation; (2) Hepatic enzymatic conversion via the kynurenine pathway (catalyzed by IDO1/2 and TDO) generates kynurenine and its downstream metabolites (kynurenic acid, 3-hydroxykynurenine, quinolinic acid). These metabolites bind to cytosolic AhR, triggering dissociation from chaperone proteins (e.g., HSP90) and nuclear translocation, where AhR forms a complex with ARNT (AhR nuclear translocator) and binds to dioxin response elements (DREs) in target gene promoters. Transcriptional activation of downstream genes mediates three key pathological processes in MASLD: (1) Upregulation of lipid synthesis genes (e.g., SREBP-1c, FASN) and fatty acid uptake-related gene (CD36) promotes hepatocyte lipid accumulation (steatosis); (2) Induction of pro-inflammatory cytokines (IL-6, TNF-α, MCP-1) and reactive oxygen species (ROS) exacerbates hepatic inflammation and oxidative stress; (3) Activation of hepatic stellate cells (HSCs) stimulates collagen deposition, leading to liver fibrosis. The synergistic effects of these processes drive the progression of MASLD from simple steatosis to steatohepatitis and fibrosis.

The tryptophan-AhR signaling pathway plays a pivotal role in regulating both immune responses and metabolic processes, which are central to the pathogenesis of MASLD (13). Tryptophan, an essential amino acid, undergoes metabolic conversion into a variety of bioactive metabolites, including kynurenine, which can activate AhR and subsequently modulate several key cellular functions (14, 15). Recent research has revealed that dysregulation of tryptophan metabolism and AhR activation is linked to numerous metabolic and inflammatory diseases, including MASLD (13). Understanding the complex interplay between tryptophan metabolism and AhR signaling is crucial for elucidating the mechanisms underlying MASLD and identifying potential therapeutic targets. In this section, we will first explore the metabolic pathways of tryptophan and its interaction with AhR, followed by a review of the current research examining the role of tryptophan-AhR signaling in metabolic diseases (see **Figure 1**).

### 2.1 Tryptophan metabolism and the AhR pathway

Tryptophan is an essential amino acid, meaning it must be obtained through the diet, as the human body cannot synthesize it (16). Beyond its well-established role as a precursor for proteins and neurotransmitters, tryptophan undergoes complex metabolic conversions that yield several bioactive metabolites (16, 17). The most notable of these is kynurenine, a key intermediate in the catabolism of tryptophan via the kynurenine pathway (16). This pathway, which is primarily mediated by the enzymes indoleamine 2,3-dioxygenase (IDO) and tryptophan 2,3-dioxygenase (TDO), plays a critical role in regulating immune responses and cellular homeostasis (18). The kynurenine pathway, activated by inflammation and oxidative stress, leads to the production of kynurenine and its downstream metabolites, such as 3-hydroxykynurenine, quinolinic acid, and kynurenic acid (17, 19). These metabolites not only influence immune cell function but also have direct effects on various physiological processes, including neuronal activity and vascular health (17, 20). Among these metabolites, kynurenine stands out due to its ability to activate the AhR, a ligand-activated transcription factor that is central to the regulation of immune function, metabolism, and homeostasis (18).

AhR, originally identified for its role in mediating the effects of environmental toxins like dioxins, has since been recognized as a crucial modulator of immune and metabolic responses (21). Upon binding to its ligands, including kynurenine, AhR translocates to the nucleus, where it regulates the expression of target genes involved in immune cell differentiation, inflammation, oxidative stress, and lipid metabolism (21–23). The activation of AhR by kynurenine and other tryptophan metabolites links the immune system and metabolic processes, making AhR an important player in both health and disease (21, 24). In the liver, AhR activation by tryptophan metabolites affects several key metabolic pathways, including lipid homeostasis, glucose metabolism, and inflammation (25). AhR signaling in hepatocytes can modulate lipid droplet formation, fatty acid oxidation, and triglyceride synthesis, which are all critical processes in the development of MASLD (26). Additionally, AhR has been shown to influence hepatic macrophage activation and cytokine production, contributing to the inflammatory milieu that exacerbates liver damage in metabolic diseases (27).

Importantly, the tryptophan-AhR signaling axis is not limited to liver function alone. It also involves interactions with the gut microbiota (e.g, *Lactobacillus*, *Bacteroidetes*, *Firmicutes*), which plays a central role in modulating tryptophan metabolism (28). Microbial enzymes can convert tryptophan into various metabolites, including indoles and indole derivatives (the main AhR ligands produced by intestinal flora), some of which also activate AhR (28). This cross-talk between the gut and liver highlights the complexity of tryptophan-AhR signaling, which can be influenced by diet, gut health, and microbial composition (29). The dysregulation of this pathway, particularly in the context of metabolic diseases like MASLD, has profound implications for disease progression (13). Altered tryptophan metabolism and aberrant AhR signaling can lead to an imbalance between pro-inflammatory and anti-inflammatory signals, promoting hepatic inflammation, steatosis, and fibrosis (13, 23). Understanding the dynamics of tryptophan metabolism and AhR activation is thus crucial for identifying therapeutic strategies that target this pathway to prevent or treat MASLD and other related metabolic disorders (30, 31).

### 2.2 Current research on tryptophan-AhR signaling in metabolic diseases

Recent research has increasingly focused on the role of tryptophan and the AhR signaling pathway in metabolic diseases, highlighting its involvement in inflammation, immune modulation, and metabolic dysfunction (see **Table 1**) (32, 33). This research has provided valuable insights into the complex mechanisms that link tryptophan metabolism to diseases such as MASLD, obesity, insulin resistance, and cardiovascular diseases (7). In the context of MASLD, several studies have demonstrated that dysregulated tryptophan metabolism, particularly the upregulation of the kynurenine pathway, plays a critical role in the disease’s progression (7). Elevated levels of kynurenine and its downstream metabolites have been associated with increased hepatic inflammation, oxidative stress, and fibrosis, all of which contribute to the pathogenesis of MASLD (34). A key mechanism appears to involve AhR activation by kynurenine, which leads to the induction of pro-inflammatory cytokines and alterations in lipid metabolism (10, 35). For instance, activation of AhR in hepatocytes has been shown to promote the accumulation of lipids and triglycerides, exacerbating steatosis and facilitating the transition from simple fatty liver to more severe forms of liver damage, such as steatohepatitis and fibrosis (22).

**Table 1:** Summary of core results on the relationship between tryptophan AhR signaling pathway and MASLD.

Research subject	Core intervention/testing	Key cells/microbiota	Core results	Research conclusion	Reference
Obesity with MASLD	Tryptophan metabolic profiling	Hepatocytes	The levels of kyrenine, a key product of tryptophan metabolism, in the peripheral blood and liver tissues of obese patients with MASLD are significantly increased, the kyrenine/tryptophan ratio rises, and the levels of indole metabolites decrease, which are positively correlated with the degree of hepatic steatosis.	Disorder of tryptophan metabolic profile is an important feature of obesity combined with MASLD. Activation of the kynurenine pathway can serve as a potential metabolic marker for liver injury in this population.	(7)
High-fat diet-induced obese mice	Gut microbiota detection + Tryptophan-AhR axis verification	Hepatocytes, kupffer cells; *Lactobacillus genus, Bacteroidetes phylum*	High-fat diet led to decreased abundance of *Lactobacillus genus*, imbalance of *Bacteroidetes phylum* ratio, reduced production of AhR ligands from intestinal tryptophan metabolism, and significantly up-regulated AhR expression and activation level in liver tissues of mice. The secretion of pro-inflammatory cytokines by kupffer cells increased, and hepatic lipid accumulation in hepatocytes aggravated. Supplement of *Lactobacillus* could restore the level of AhR ligands, inhibit hepatic AhR activation and improve hepatic steatosis.	The disorder of intestinal microbiota structure activates the hepatic tryptophan-AhR axis by down-regulating the production of tryptophan-derived AhR ligands, mediates abnormal lipid metabolism in hepatocytes and inflammatory activation of kupffer cells, which is an important mechanism of high-fat diet-induced MASLD in mice.	(13)
MASH cellular/mice models	AhR inhibitor intervention + Ferroptosis marker detection	Hepatocytes	AhR inhibitors could significantly reduce the expression of ferroptosis-related markers (e.g., PTGS2, ACSL4) in hepatocytes of MASH models, decrease the accumulation of reactive oxygen species and lipid peroxidation in hepatocytes, and alleviate hepatocellular injury and the degree of hepatic inflammation; AhR overexpression could aggravate hepatocellular ferroptosis and MASH pathological changes.	AhR activation promotes hepatocellular injury and inflammatory progression of MASH by mediating ferroptosis of hepatocytes, and inhibition of AhR signaling can be a potential target for MASH treatment.	(12)
Metabolic syndrome mice/humans	Gut microbiota AhR ligand detection	Intestinal epithelial cells and hepatocytes; *Firmicutes*/*Bacteroidetes*	The ratio of *Firmicutes*/*Bacteroidetes* in the intestine of mice and humans with metabolic syndrome was increased, the level of indole-derived AhR ligands produced by intestinal microbiota was significantly decreased, the integrity of intestinal epithelial barrier was damaged, the expression of inflammatory factors in liver tissues was increased, and hepatic steatosis and insulin resistance were aggravated. Exogenous supplement of AhR ligands could repair the intestinal barrier and improve hepatic insulin resistance and steatosis.	The deficiency of gut microbiota-derived AhR ligands destroys the gut-liver axis homeostasis, promotes hepatic steatosis and insulin resistance related to metabolic syndrome. Targeted regulation of intestinal microbiota to increase AhR ligand production can improve the metabolic disorder of the gut-liver axis.	(37)
MASLD mice models	3,3’-Diindolylmethane/Cinnabarinic acid intervention	Hepatocytes, HSCs	3,3’-Diindolylmethane and cinnabarinic acid could up-regulate the phosphorylation level of AMPK by activating AhR signaling, inhibit the expression of SREBP-1c and lipid synthesis in hepatocytes, and simultaneously inhibit the activation of HSCs and collagen secretion, thus significantly alleviating the degree of hepatic steatosis and liver fibrosis in mice.	AhR-specific ligands such as 3,3’-diindolylmethane and cinnabarinic acid can exert anti-MASLD and anti-fibrotic effects by regulating lipid metabolism in hepatocytes and activation of HSCs.	(41)
Diet-induced obese mice	AhR inhibitor intervention	Hepatocytes	AhR inhibitors could significantly inhibit the expression of lipid metabolism-related genes such as SREBP-1c and PPAR-γ in hepatocytes of obese mice, reduce fatty acid synthesis and lipid storage in hepatocytes, increase the expression of genes related to mitochondrial fatty acid oxidation, and improve hepatic steatosis and systemic insulin resistance.	Inhibition of AhR signaling can reverse diet-induced hepatic steatosis in mice by remodeling the lipid metabolism balance of hepatocytes, which is an effective strategy for improving obesity-related MASLD.	(42)
Hepatic fibrosis mice models	AhR signaling + HSCs activation assay	HSCs	The expression and activation level of AhR in liver tissues of mice with liver fibrosis were significantly increased, which was positively correlated with the expression of HSCs activation markers (α-SMA, Col1a1). AhR knockout could inhibit the expression of pro-fibrotic factors such as TGF-β and PDGF in HSCs, reduce the activation of HSCs and extracellular matrix deposition, and alleviate liver fibrosis.	AhR signal activation promotes the process of liver fibrosis by mediating the activation of HSCs and the secretion of pro-fibrotic factors. Targeted inhibition of AhR signal in HSCs can delay the development of MASLD-related liver fibrosis.	(45)
Children/adolescents with obesity-related MASLD	Peripheral blood tryptophan derivative detection	Hepatocytes	The levels of kynurenine pathway products such as kynurenine and 3-hydroxykynurenine in the peripheral blood of children and adolescents with obesity-related MASLD were increased, while the levels of microbiota-derived tryptophan derivatives such as indole-3-propionic acid were decreased, and these changes were significantly correlated with the levels of liver enzymes (ALT/AST) and the degree of hepatic steatosis.	Peripheral blood tryptophan derivative profile can be used as a non-invasive diagnostic and disease assessment marker for children and adolescents with obesity-related MASLD, and tryptophan metabolic disorder is involved in the pathogenesis of MASLD in children and adolescents.	(46)

In addition to its effects in the liver, recent studies have highlighted the role of tryptophan-AhR signaling in regulating systemic metabolic processes. For example, research has demonstrated that AhR activation in adipocytes influences fat storage and insulin sensitivity, suggesting that tryptophan metabolites may also contribute to the development of obesity and insulin resistance (36). This is supported by animal studies showing that modulation of the tryptophan-AhR axis can alter adiposity and glucose homeostasis (37, 38). Furthermore, the interaction between AhR signaling and gut microbiota has emerged as a significant factor in metabolic regulation. The gut microbiota can influence tryptophan metabolism by producing metabolites that activate AhR, thereby linking the gut-liver axis to systemic metabolic health (28).

One notable area of research is the potential for dietary interventions to modulate the tryptophan-AhR pathway and improve metabolic outcomes. Studies have shown that diet-induced changes in tryptophan intake or the gut microbiome can influence AhR activation, offering a potential strategy for managing metabolic diseases (39). For example, a diet rich in prebiotics or probiotics can alter gut microbiota composition, leading to changes in tryptophan metabolism and subsequent modulation of AhR signaling (40). These findings suggest that dietary strategies aimed at optimizing tryptophan metabolism could be a promising therapeutic approach for preventing or treating MASLD and other metabolic disorders (41).

Moreover, research is increasingly focusing on pharmacologically modulating the tryptophan-AhR signaling pathway. Studies have explored using AhR antagonists to influence this pathway in metabolic disease models (12, 42). Kynurenine analogs and AhR-specific ligands have shown potential in modulating immune responses and reducing inflammation in animal models (43). However, targeting AhR therapeutically is complex due to its dual role as both a pro-inflammatory and anti-inflammatory mediator, with effects varying by tissue and disease context (44). While much progress has been made in understanding the role of tryptophan-AhR signaling in metabolic diseases, several questions remain. Future research will need to address the long-term effects of modulating this pathway, explore the interactions between tryptophan metabolites and other signaling pathways, and identify reliable biomarkers for assessing AhR activation in clinical settings. With these efforts, the tryptophan-AhR axis could become a key target for the development of novel therapeutic strategies for MASLD and related metabolic conditions.

## 3 The role of tryptophan-AhR signaling in the progression of MASLD

**Figure 2:**
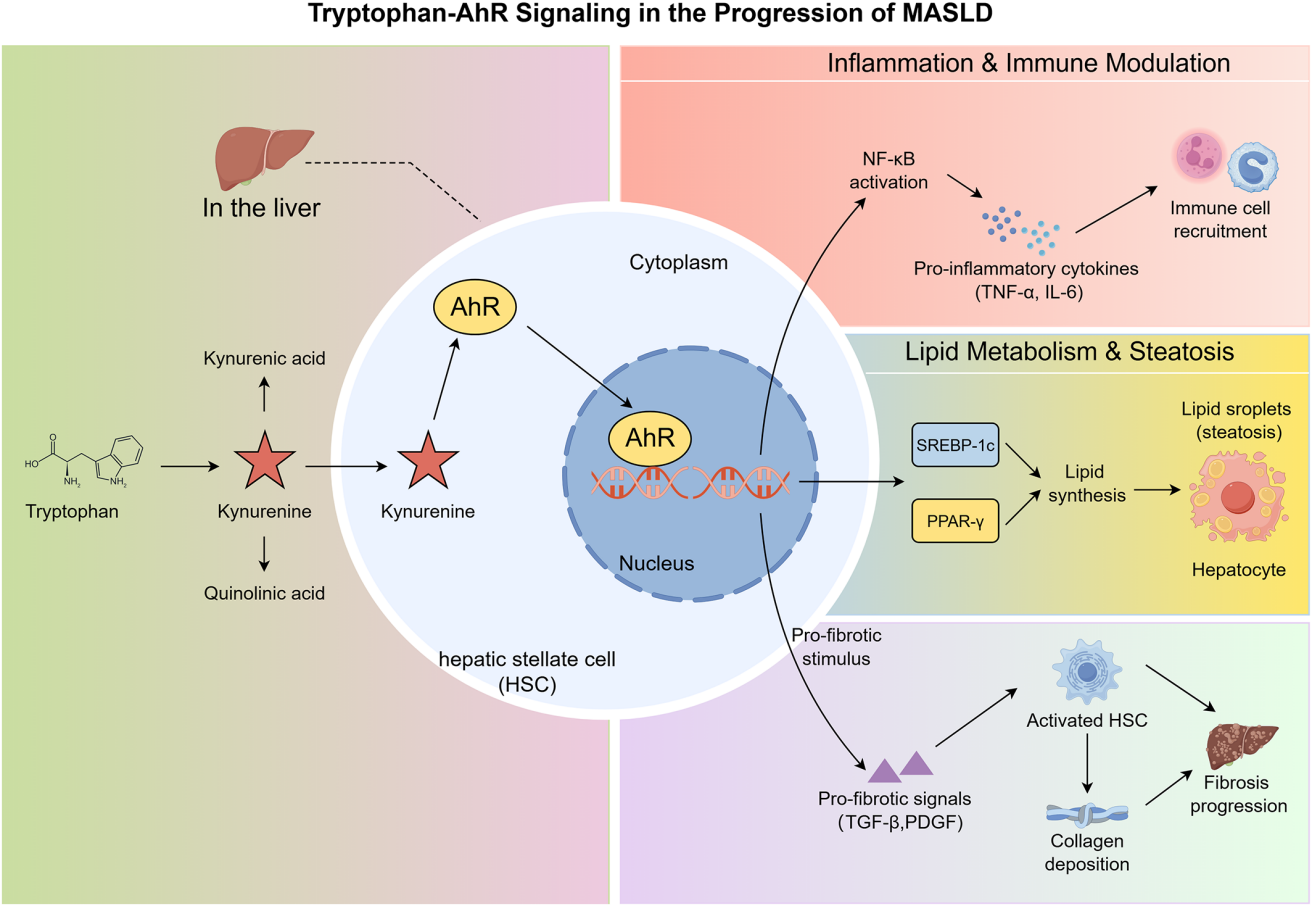
Mechanisms by which the tryptophan-AhR signaling pathway drives MASLD progression (this figure was created by Figdraw). Tryptophan is metabolized by the enzymes IDO or TDO into kynurenine, a potent endogenous ligand for the aryl hydrocarbon receptor (AhR). Upon ligand binding, cytosolic AhR translocates into the nucleus to modulate gene transcription, promoting MASLD pathology through three distinct axes: (1) AhR activation induces NF-κB signaling, triggering the release of pro-inflammatory cytokines (TNF-α, IL-6). These cytokines recruit immune cells (e.g., macrophages, neutrophils) to the liver, amplifying the inflammatory cascade and facilitating the transition from steatosis to steatohepatitis; (2) AhR upregulates the expression of transcription factors SREBP-1c and PPAR-γ. SREBP-1c promotes *de novo* fatty acid and triglyceride synthesis, while PPAR-γ enhances lipid storage, leading to excessive lipid droplet accumulation in hepatocytes (steatosis). Additionally, AhR impairs mitochondrial fatty acid oxidation, further exacerbating lipid buildup; (3) Persistent hepatic inflammation and AhR-mediated secretion of pro-fibrotic signals (TGF-β, PDGF) activate hepatic stellate cells (HSCs), which transdifferentiate into myofibroblasts. These cells produce excessive extracellular matrix components (e.g., collagen), resulting in hepatic fibrosis. Collectively, these three axes form a pathological network that drives the stepwise progression of MASLD.

The progression of MASLD is characterized by a series of complex events, beginning with hepatic fat accumulation and potentially advancing to more severe forms of liver injury such as steatohepatitis, fibrosis, and even hepatocellular carcinoma (47). While the exact molecular mechanisms driving these processes are still being elucidated, the role of tryptophan metabolism and the AhR signaling pathway has emerged as a crucial component in the progression of MASLD (7, 48). This section will explore how the tryptophan-AhR axis contributes to the pathogenesis of MASLD, focusing on three key aspects: hepatic inflammation, lipid metabolism, and fibrosis progression (see **Figure 2**).

### 3.1 Hepatic inflammation

Inflammation is a hallmark of MASLD, particularly as the disease progresses to more severe stages such as steatohepatitis (13). In the early stages of MASLD, excess lipid accumulation in hepatocytes induces a pro-inflammatory environment (49). This inflammatory response is driven by various factors, including oxidative stress, cytokine release, and activation of the innate immune system (50–52). The tryptophan-AhR signaling pathway plays a significant role in modulating these inflammatory processes (51, 53).

AhR activation, particularly through the binding of tryptophan metabolites like kynurenine, has been shown to promote the expression of key inflammatory cytokines, such as tumor necrosis factor-alpha (TNF-α), interleukin-6 (IL-6), and interleukin-1 beta (IL-1β), which are crucial for the progression of liver inflammation (21, 54). In hepatocytes, AhR signaling triggers the activation of nuclear factor-kappa B (NF-κB), a central regulator of inflammation (54, 55). This activation leads to the production of pro-inflammatory cytokines, which amplify the inflammatory response and recruit immune cells such as macrophages and neutrophils (macrophages are the main immune cells involved in AhR-mediated liver inflammation) to the site of injury (21, 52).

Furthermore, the activation of AhR in liver-resident macrophages, known as Kupffer cells, has been shown to enhance the secretion of inflammatory mediators (56). The interplay between hepatocytes and macrophages via the AhR signaling axis exacerbates the inflammatory response, contributing to the transition from simple steatosis to steatohepatitis, a more aggressive form of liver injury (56, 57). Chronic inflammation, in turn, accelerates liver damage and fibrosis, further complicating the pathogenesis of MASLD (56–58).

### 3.2 Lipid metabolism

Lipid metabolism is another crucial aspect of MASLD progression, with dysregulated lipid accumulation in hepatocytes being a primary feature of the disease (59). Tryptophan metabolites, particularly kynurenine, have been implicated in altering lipid metabolism in the liver through AhR activation (22).

In hepatocytes, upon AhR activation by kynurenine, downstream effects include the modulation of genes involved in lipid homeostasis, including sterol regulatory element-binding protein 1c (SREBP-1c) and peroxisome proliferator-activated receptor gamma (PPAR-γ) (25, 60). These transcription factors are essential for regulating lipid synthesis and storage in hepatocytes (61, 62). SREBP-1c, in particular, promotes the synthesis of fatty acids and triglycerides, while PPAR-γ regulates adipogenesis and lipid storage (61, 62). When AhR signaling is aberrantly activated, as seen in MASLD, the upregulation of these pathways leads to increased lipid synthesis and accumulation in the liver, further exacerbating steatosis (25, 60).

Moreover, AhR activation can impair lipid oxidation pathways, which are essential for maintaining lipid balance in hepatocytes (63). By inhibiting the expression of genes involved in mitochondrial fatty acid oxidation, AhR signaling favors lipid storage over metabolism, promoting the development of hepatic steatosis (63, 64). This dysregulated lipid metabolism is one of the key factors that leads to the progression of MASLD from simple steatosis to more advanced stages such as steatohepatitis and fibrosis (63, 64).

In addition, the AhR pathway interacts with other metabolic regulators, such as AMP-activated protein kinase (AMPK), which is known to play a central role in energy balance and lipid metabolism (65, 66). Under conditions of metabolic stress, AMPK activation inhibits lipid accumulation and promotes fatty acid oxidation (67). However, through its regulatory effects on AhR, the dysregulated AhR signaling pathway can impair AMPK activation, further exacerbating lipid buildup in hepatocytes (65, 66).

### 3.3 Fibrosis progression

The progression of MASLD to fibrosis and cirrhosis is a critical aspect of the disease’s clinical course and a key factor contributing to liver-related morbidity and mortality (68). The role of tryptophan-AhR signaling in the development of fibrosis has been an area of active research (69). AhR signaling has been shown to influence the activation of hepatic stellate cells (HSCs), which are the primary effector cells in liver fibrosis (45).

HSCs, when activated by pro-fibrotic signals, transdifferentiate into myofibroblasts that produce extracellular matrix proteins, including collagen, which are key components of fibrotic tissue (70). Recent studies suggest that AhR activation plays a role in the activation of HSCs by promoting the expression of pro-fibrotic factors such as transforming growth factor-beta (TGF-β) and platelet-derived growth factor (PDGF) (the key factors for HSCs activation in MASLD fibrosis) (71). These factors contribute to the excessive deposition of collagen and other matrix proteins, leading to fibrosis (70).

Additionally, the chronic inflammation mediated by AhR signaling in the liver contributes to the activation of HSCs (72). As inflammatory cytokines like TNF-α and IL-6 are released from macrophages and hepatocytes, they promote the activation of HSCs and the production of extracellular matrix components (72). The persistent inflammatory environment, driven in part by the tryptophan-AhR axis, thus accelerates the progression from steatosis to fibrosis, and ultimately to cirrhosis in severe cases of MASLD (72, 73).

## 4 Challenges and limitations

The exploration of tryptophan-AhR signaling in MASLD presents several significant challenges that need to be addressed for a comprehensive understanding of its role in disease progression. One of the primary difficulties lies in the complexity of AhR signaling itself. As a ligand-activated transcription factor, AhR regulates a wide array of biological processes, including immune responses, metabolism, and cell differentiation, with both pro-inflammatory and anti-inflammatory effects depending on the context (12, 45). This duality complicates the interpretation of its role in MASLD, as its activation may either exacerbate or alleviate liver injury, depending on the stage of the disease and the cell types involved (26, 45, 74). Additionally, the tissue-specific effects of AhR activation add another layer of complexity (12, 45). In the liver, AhR has different impacts in hepatocytes, Kupffer cells, and HSCs, which makes it difficult to draw generalized conclusions about its role in MASLD. The same tryptophan metabolites that activate AhR may trigger diverse responses in different liver cell types, further complicating the overall understanding of its contribution to the disease (75, 76). Furthermore, the variability in tryptophan metabolism itself is another significant challenge. Diet, gut microbiota composition, and genetic factors can all influence tryptophan metabolism, leading to different levels of tryptophan metabolites, such as kynurenine (15). This variability complicates efforts to standardize treatment approaches or predict how individuals may respond to modulation of the tryptophan-AhR axis (37). Finally, AhR signaling does not function in isolation; it interacts with various other metabolic and inflammatory pathways, such as NF-κB, PPARs, and AMPK (26, 60). These complex interactions make it difficult to isolate the specific contributions of AhR signaling to MASLD and complicate the development of targeted therapeutic strategies.

In addition to these challenges, several limitations exist in the current research that hinder the ability to fully characterize the role of tryptophan-AhR signaling in MASLD. Most studies on this pathway are either cross-sectional or short-term in nature, focusing on specific stages of the disease, which limits our understanding of the long-term effects of tryptophan-AhR modulation (77). Longitudinal studies tracking the progression of MASLD in response to changes in tryptophan metabolism and AhR activity are needed to determine whether altering this pathway could provide lasting therapeutic benefits. Moreover, much of the existing research is based on animal models or *in vitro* studies, which, while valuable, may not fully replicate the complexity of MASLD in humans (12, 26). The differences between species, as well as the influence of human-specific factors such as diet and genetic diversity, underscore the need for more human-based studies to confirm the findings observed in animal models (52). Additionally, while AhR is a promising therapeutic target, its modulation presents significant challenges. The pathway’s pleiotropic effects, combined with the potential for off-target consequences, make it difficult to develop drugs that specifically target AhR without unintended effects (78). The dual nature of AhR signaling, with its ability to both promote inflammation and resolution, further complicates the design of therapies that can effectively regulate its activity in the context of MASLD. Finally, the heterogeneity of MASLD itself poses a limitation, as the disease varies widely in its severity and progression across individuals. The association of MASLD with other comorbidities such as obesity, diabetes, and dyslipidemia makes it difficult to adopt a one-size-fits-all approach to treatment, and the variability in how patients respond to changes in tryptophan metabolism and AhR signaling presents a significant obstacle in applying this knowledge to clinical settings.

In summary, the study of tryptophan-AhR signaling in MASLD offers valuable insights but faces challenges due to the complexity of AhR signaling, tissue-specific effects, and individual differences in tryptophan metabolism. Current research limitations, including a lack of longitudinal human studies and challenges in targeting AhR therapeutically, underscore the need for more robust data and innovative treatments. Overcoming these hurdles is essential for developing practical therapies for MASLD and other metabolic disorders.

## 5 Future directions

Despite the challenges and limitations associated with studying tryptophan-AhR signaling in MASLD, the growing body of evidence suggests that this pathway holds significant therapeutic potential. Future research should focus on several key areas to further elucidate the role of tryptophan-AhR signaling in MASLD and explore its clinical applications.

One promising direction is the development of longitudinal studies in human populations. While animal models have provided valuable insights, human-based studies are crucial to understanding how tryptophan metabolism and AhR activation contribute to the progression of MASLD over time (9, 79). These studies could also help identify biomarkers that predict disease progression and response to treatments targeting the tryptophan-AhR pathway (7, 24, 46). Such biomarkers could be used for patient stratification, ensuring that therapies are tailored to individuals based on their unique metabolic profiles.

Another critical area of research is the identification and optimization of therapeutic agents targeting the tryptophan-AhR signaling axis. Given the complexity of AhR signaling, there is a need for more specific modulators of this pathway that can selectively activate or inhibit AhR depending on the context of the disease (80). Drug development efforts should focus on small molecules or biologics that can either enhance the protective effects of AhR activation in the liver or suppress its pro-inflammatory and pro-fibrotic effects, particularly in advanced stages of MASLD (12, 45). Additionally, dietary interventions that modify tryptophan metabolism or gut microbiota composition may offer a non-invasive approach to modulating AhR activity. Prebiotic or probiotic therapies, as well as dietary modifications, could alter the levels of tryptophan metabolites, thus influencing AhR activation in a way that benefits metabolic health (81).

Finally, exploring the interactions between AhR signaling and other metabolic and immune pathways will be crucial for developing integrative therapeutic strategies. Understanding how AhR interacts with pathways like NF-κB, PPARs, and AMPK in MASLD will provide a more comprehensive approach to treating the disease. By targeting multiple interconnected pathways, it may be possible to achieve more effective and sustainable treatments for MASLD.

## 6 Discussion

In conclusion, tryptophan-AhR signaling constitutes a pivotal yet intricate pathway in the pathogenesis of MASLD, affecting inflammation, lipid metabolism, and fibrosis progression. The dual nature of AhR activation, which exhibits both pro-inflammatory and anti-inflammatory effects, complicates the understanding of its precise role in disease progression. Although significant advancements have been made in elucidating the effects of tryptophan metabolites on liver function, critical questions remain regarding their long-term impact and therapeutic potential. The variability in tryptophan metabolism among individuals and the tissue-specific effects of AhR activation underscore the necessity for more personalized and targeted therapeutic approaches. Future research should prioritize longitudinal human studies and the development of specific AhR modulators and dietary interventions, which will be essential in harnessing the full potential of tryptophan-AhR signaling in MASLD treatment. Ultimately, sustained investigation into this pathway could lead to the development of novel and more effective strategies for managing MASLD and related metabolic disorders.

## Data Availability

The original contributions presented in the study are included in the article/supplementary material. Further inquiries can be directed to the corresponding author.

## Glossary

MASLD: metabolic dysfunction-associated steatotic liver disease
NAFLD: non-alcoholic fatty liver disease
MASH: metabolic dysfunction-associated steatohepatitis
NASH: non-alcoholic steatohepatitis
AhR: aryl hydrocarbon receptor
IDO: indoleamine 2,3-dioxygenase
TDO: tryptophan 2,3-dioxygenase
PTGS2: prostaglandin-endoperoxide synthase 2
ACSL4: acyl-CoA synthetase long-chain family member 4
α-SMA: alpha-smooth muscle actin
Col1a1: collagen type I alpha 1
ALT: alanine aminotransferase
AST: aspartate aminotransferase
TNF-α: tumor necrosis factor-alpha
IL-6: interleukin-6
IL-1β: interleukin-1 beta
NF-κB: nuclear factor-kappa B
SREBP-1c: sterol regulatory element-binding protein 1c
PPAR-γ: peroxisome proliferator-activated receptor gamma
AMPK: AMP-activated protein kinase
HSCs: hepatic stellate cells
TGF-β: transforming growth factor-beta
PDGF: platelet-derived growth factor
